# Supplementary material for: Causal interplay between lactose intolerance and gut microbiota: a combined bidirectional Mendelian randomization and in vivo validation study
Source: Front Nutr. 2026 Jun 1;13:1803337. doi: 10.3389/fnut.2026.1803337 (PMC13265576; doi:10.3389/fnut.2026.1803337)
Supplement: Supplementary file 1 [file Data_Sheet_1.zip › supplementary materials/Forward/leave one out cross validation/ebi-a-GCST90027597.finngen_R12_E4_LACTONAS.pdf]

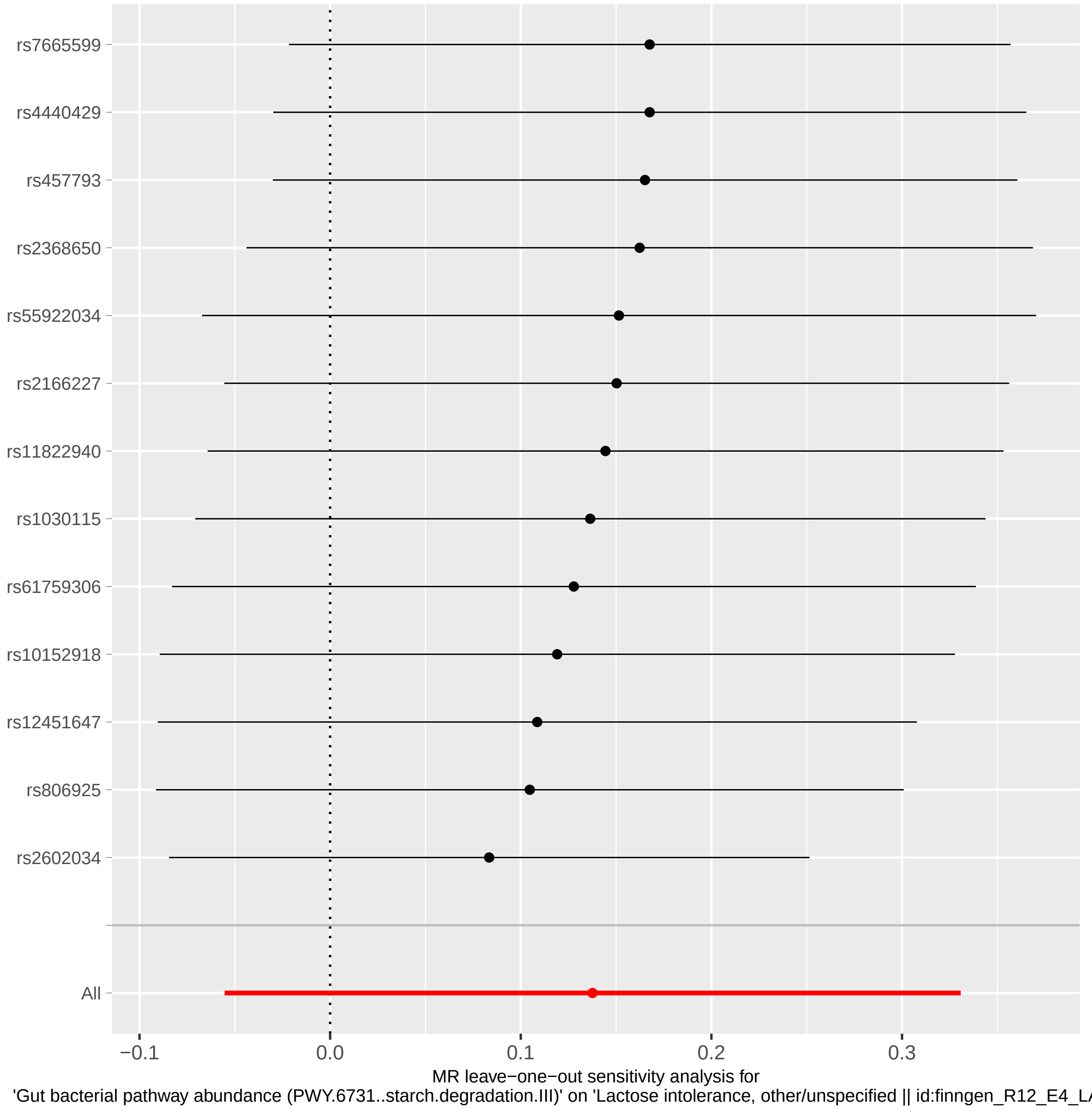

rs7665599
rs4440429
rs457793
rs2368650
rs55922034
rs2166227
rs11822940
rs1030115
rs61759306
rs10152918
rs12451647
rs806925
rs2602034
All
−0.1
0.0
0.1
0.2
0.3
MR leave−one−out sensitivity analysis for
'Gut bacterial pathway abundance (PWY.6731..starch.degradation.III)' on 'Lactose intolerance, other/unspecified || id:finngen_R12_E4_L
